# Retreatment rate and strategies for recurrent and residual aneurysms after Woven EndoBridge (WEB) treatment: a comprehensive systematic review and meta-analysis

**DOI:** 10.1007/s10143-025-03532-y

**Published:** 2025-05-02

**Authors:** Ali Mortezaei, Forough Yazdanian, Mohammad Mirahmadi Eraghi, Farid Qoorchi Moheb Seraj, Romulo Augusto Andrade de Almeida, Parsa Saberian, Mohammad Amin Habibi, Justin H. Granstein, Humain Baharvahdat, Redi Rahmani, Robert M. Starke

**Affiliations:** 1 Gonabad University of Medical Sciences, Gonabad, Iran; 2 Neurosurgical Service, Beth Israel Deaconess Medical Center, Boston, MA USA; 3 School of Medicine, Islamic Azad University, Qeshm International Branch, Qeshm, Iran; 4 Neurosurgical Department, Neurovascular Section, Ghaem Hospital, Mashhad University of Medical Sciences, Mashhad, Iran; 5 Department of Neurosurgery, The University of Texas MD Anderson Cancer Center, Houston, TX USA; 6 Faculty of Medicine, Hormozgan University of Medical Sciences, Bandar Abbas, Iran; 7 Department of Neurosurgery, Shariati Hospital, Tehran University of Medical Sciences, Tehran, Iran; 8 Department of Interventional Neuroradiology, Rothschild Foundation Hospital, Paris, France; 9 Department of Neurosurgery, Perelman School of Medicine, University of Pennsylvania, Philadelphia, PA USA; 10 Department of Neurological Surgery, Radiology, Neurosciences, Pharmacology, University of Miami School of Medicine, Miami, FL USA

**Keywords:** Woven EndoBridge, Intrasaccular Flow Diverter, Retreatment, Aneurysm

## Abstract

Woven Endo Bridge (WEB) is a flow diverter device used to treat wide-necked intracranial aneurysms. We conducted a systematic review and meta-analysis to determine the retreatment rate and strategies following aneurysmal recurrence after WEB treatment. A systematic literature search was conducted through four electronic databases. The Cochrane risk of bias tool for non-randomized trials (ROBINS-I) was used for risk of bias assessment. A meta-analysis and meta-regression were performed on relevant variables. A total of 29 studies with 2067 patients were included. Initially treated aneurysms had an adequate occlusion rate of 84.7% (20 studies, 980/1156, 95%CI: 79%—89%) at the last follow-up. The overall retreatment rate was 8.6% (24 studies, 251/2893, 95%CI: 6.5%—10.9%). Following retreatment, the immediate complete occlusion rate post retreatment and complete occlusion rate at last follow-up were 52% (six studies, 95%CI, 11%—91%) and 65% (six studies, 77/120, 95%CI: 56%—73%) respectively. Stent-assisted coiling achieved a 100% (95%CI: 57%—100%, P-value < 0.01) immediate adequate occlusion rate and 74% complete occlusion rate (95%CI: 16%—100%, *P*-value = 0.63) at last follow-up, which was higher than clipping, coiling, and flow diversion. On meta-regression, aneurysm height and neck width significantly associated with higher retreatment rate. The current meta-analysis showed a 9% retreatment rate after initial aneurysms treatment with WEB and overall adequate retreatment occlusion was high. Although stent-assisted coiling showed a higher adequate occlusion rate post-operatively, there was no significant difference between retreatment strategies in long-term radiological outcomes.

**Supplementary Information:**

The online version contains supplementary material available at 10.1007/s10143-025-03532-y.

## Introduction

Woven Endo Bridge (WEB) is a flow disruptor device represents a safe option for the treatment of wide-necked intracranial aneurysms [1]. The intravascular deployment of a WEB device has made it an alternative to the embolization of ruptured aneurysms due to a reduction in the need for an antiplatelet therapy regimen [2, 3]. However, the complete occlusion rate in ruptured aneurysms is about 43% [4] compared to the 96% rate in microsurgical clipping [5]. Although some studies suggested that in short- and long-term follow-up, 5–12% of patients may require retreatment [6, 7] following WEB treatment, retreatment rate following microsurgery and endovascular strategies were 3.8% and 8.8%, respectively [8].

In the setting of retreatment, both microsurgery and endovascular treatment (including, coiling, stent-assisted coiling, and flow diverters) have been reported as options [9, 10]. The factors associated with aneurysm recurrence and optimal management of aneurysm recanalization following WEB treatment are not fully elucidated. Identifying these factors is crucial for defining an appropriate treatment strategy and improving long-term outcomes [11, 12].

In the current systematic review and meta-analysis, we aimed to investigate outcomes following the initial WEB deployment, location of the initially treated and retreated aneurysms, factors associated with retreatment rate, occlusion rate following retreatment and based on each retreatment modality, and recurrence-related to WEB type.

## Methods

### Search strategy

**Fig. 1:**
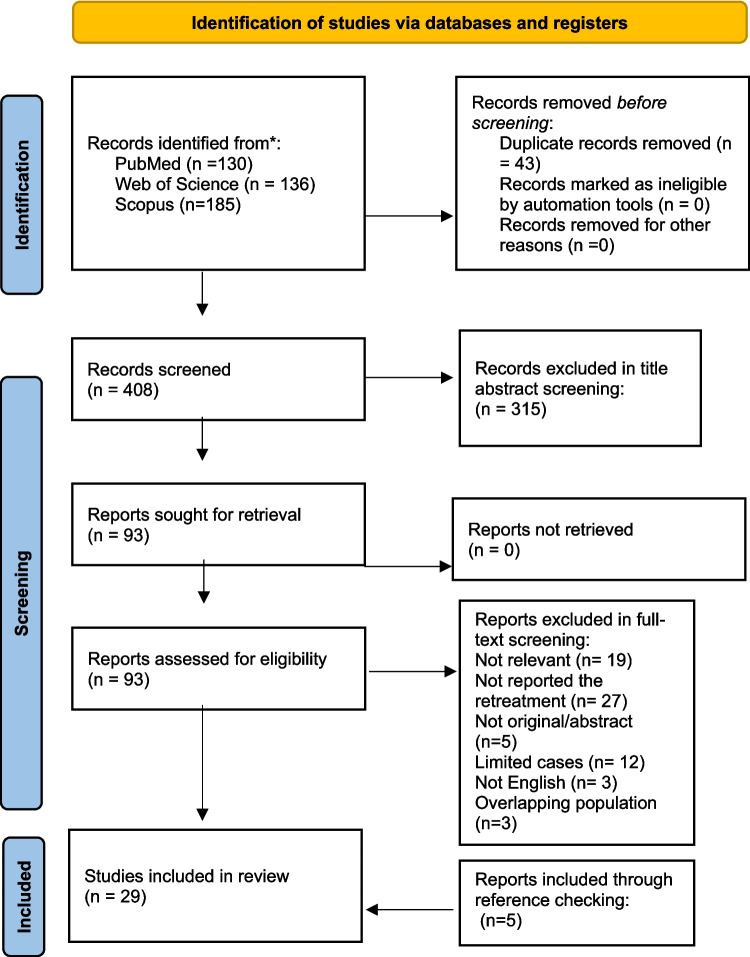
PRISMA flow chart for study selection

A systematic literature search was conducted following Preferred Reporting Items for Systematic Reviews and Meta-Analyses (PRISMA) guidelines Fig. 1. We searched electronic databases including PubMed/MEDLINE, Scopus, and Web of Science up to May 16 2024. Search strategy combined terms related to “Retreatment”, “Recurrence”, “Brain Aneurysm”, and “Woven Endobridge”. Searches were explicitly adapted for each electronic database, employing a broad selection of keywords. Complete search strategy is available in Supplement Table 1.

### Eligibility criteria and outcomes

We included studies 1) English language articles, and 2) reported at least one of the outcomes of interest. Studies were excluded if 1) they did not assess at least one of the outcomes of interest, 2) had less than 5 cases, 3) editorial, reviews, comments, letters, and poster abstracts. The primary outcomes were initially-treated and retreated complete (Raymond–Roy occlusion classification (RROC) = 1 or WEB Occlusion Scale (WOS) = A-B) [13] or adequate (RROC ≤ 2 or WOS = A-C) [13] occlusion rate, and rate of retreatment. Secondary outcomes included retreatment rate, retreatment location, and retreatment strategies.

We performed a univariate meta-regression analysis to evaluate potential influence of patient demographics (gender, age), clinical characteristics (number of patients, number of aneurysms), aneurysm-specific features (location, morphology—height, neck width, dome-to-neck ratio, partially thrombosed, rupture status), and device-related factors (WEB device types: SL, SLS, DL; and device size) on occlusion status and retreatment rate and to identify source of heterogeneity.

### Screening process and data extraction

Three authors independently screened titles and abstracts, with full texts retrieved for eligibility assessment through a blinded review process. Any discrepancies were resolved through consensus or, when necessary, by consulting senior author. Data extraction was performed using a standardized form, which included characteristics, radiological outcomes, and complications. Subsequent initial data extraction process, accuracy and resolving conflicts was conducted by a third author.

### Quality and risk of bias assessment

Two authors independently assessed quality of included studies using Cochrane risk of bias tool for non-randomized trials (ROBINS-I). Any disparity was resolved through consensus. Publication bias was assessed through visual inspection of Contour-enhanced funnel plot.

### Statistical analysis

We calculated outcomes by measuring proportions and 95% confidence intervals (CIs) for each study using a random-effects model due to expected clinical and methodological heterogeneity. For assessing statistical heterogeneity, Cochran’s Q test with a significance level (α) below 0.1 or I^2^ > 40% was considered as significant heterogeneity [14]. Furthermore, a univariate meta-regression was performed to find source of heterogeneity and to evaluate association between demographics and aneurysm morphology and initial treatment adequate occlusion at last follow-up and retreatment rate. To maintain adequate statistical power [14], meta-regression was performed for outcomes reported by at least seven studies or more. Publication bias was evaluated by visual inspection of contour-enhanced funnel plot and further confirmation by Egger’s regression test, in which a *p*-value < 0.05 was defined statistically significant. All *p*-values in hypothesis testing were two-sided, with statistical significance set at *p*-value < 0.05 unless otherwise specified. All statistical analysis was completed utilizing the “netmeta” package on R version 4.3.0.

## Results

### Study selection

451 studies were found in our systematic search. 93 studies qualified for full-text screening after passing title/abstract screening. We performed a manual check of cited references of each of the included studies to find studies missed by automatic search. Furthermore, studies with overlapping population were excluded [15–17]. 29 studies were included in final examination and qualified for quantitative synthesis following full-text screening. There was considerable reliability for study selection for both the title/abstract (Cohen’s κ = 0.91) and the full-text screening steps (Cohen’s κ = 0.95). The research selection process flowchart is displayed in PRISMA flow diagram in Fig. 1.

### Study characteristics

Detailed characteristics of included studies are outlined at Table 1. We included 2067 patients (2089 aneurysms) patients from 29 studies [3, 4, 7, 9, 10, 12, 13, 15, 18–37] that evaluated the WEB device in treatment of patients with brain aneurysms. Majority of aneurysms initially treated with WEB were unruptured. The middle cerebral artery was the most common initially treated and retreated artery. Multilobulated retreated aneurysms was 31.1% (14/45) in three studies [7, 10, 18]. The pooled demographic and baseline characteristics of included patients are shown in Table 2. The proportion of retread aneurysms with partially thrombosis [10, 18, 24, 27–29] was 3.8% (16/363, 95%CI: 0.11%− 10.7%). The rebleeding rate [3, 4, 7, 10, 18, 26, 28] after initial treatment with WEB was 0.2% (2/996).

**Table 1:** Characteristics of included studies

Study	Country	Year	Design	No of Patients	Females	No of aneurysms	Age		Reason for retreatment	Time of follow-up	Initial WEB size led to retreatment (mm)
							Mean	SD			
Abbas et al. 2022 [10]	USA	2021	Retrospective	7	4	7	58.1	17.22	Residual = 4, recurrence = 3	5 years	8 × 4 = 2, 7 × 3 = 1, 10 × 5 = 1, 6 × 3 = 1, 4 × 3 = 1, 9 × 4 = 1
Alpay et al. 2022 [18]	Finland	2022	Retrospective	66	48	66	55.6	7.45	Aneurysm remnant	24 months	10 × 6 = 1, 5 × 3 = 1, 5 × 4 = 1, 11 × 8 = 1, 6 × 3 = 1, 9 × 7 = 1, 8 × 4 = 1, 7 × 3 = 1
Arthur et al. 2019 [19]	USA	2019	Prospective	150	NA	150	NA	NA	Aneurysm remnants and recurrences	12 months	NA
Cagnazzo et al. 2023 [38]	USA	2023	Retrospective	104	79	104	63	12.02	NA	NA	NA
Caroff et al. 2023 [20]	USA	2022	Prospective	57	36	57	54	10.8	Aneurysm remnants and recurrences	Mean = 21.2 months	NA
Clajus et al. 2017 [21]	USA	2017	Prospective	108	82	114	55.6	NA	Aneurysm remnant	Mean = 13.4 months	NA
Cherian et al. 2021 [37]	USA	2021	Retrospective	91	72	91	NA	NA	Aneurysm remnants and recurrences	Mean = 8 months	NA
Cognard et al. 2015 [22]	France	2014	Retrospective	15	NA	15	53.18	7.01	NA	NA	NA
Fiorella et al. 2023 [13]	USA	2023	Prospective	150	NA	150	NA	NA	Aneurysm remnants and recurrences	5 years	NA
Fujimoto et al. 2020 [23]	USA	2020	Retrospective	41	32	41	63.5	7.8	Residual aneurysm or neck	Mean = 35.8 months	NA
Goertz et al. 2022 [12]	Germany	2022	Retrospective	182	129	182	58.3	11.9	Aneurysm remnants	12 months	NA
Kabbasch et al. 2019 [24]	Germany	2018	Retrospective	121	81	122	58.5	11.9	Recurrent (device migration = 6 and WEB compression = 4) and residual aneurysms	Mean = 6.73 months	6 × 4 = 1, 11 × 9 = 2, 5 × 3 = 2, 8 × 6 = 2, 7 × 6 = 2, 8 × 5 = 1, 14 × 12 = 1, 9 × 6 = 1, 6 × 3 = 1, 8 × 3 = 1
Khalid et al. 2019 [25]	Norway	2018	Retrospective	16	9	16	59	9.04	Increasing circulation inside aneurysm neck = 6, along WEB (between WEB and aneurysm wall) = 2, and along the device = 1	40 months	9 × 7 = 3, 11 × 9 = 1, 9 = 1, 8 × 4 = 1, 14 × 11 = 1, 9 × 6 = 1,
Kranawetter et al. 2023 [9]	Germany	2023	Retrospective	5	4	5	57.6	5.75	NA	NA	NA
Kortman et al. 2023 [3]	Netherland	2023	Prospective	100	57	100	58.5	NA	Recurrent and residual aneurysms	5 years	4 = 1, 8 = 1, 9 × 4 = 1, 11 = 1, 7 × 4 = 1
Lawson et al. 2017 [26]	United Kingdom	2016	Retrospective	25	18	25	57.3	NA	Aneurysm recurrence	≥ 3 months	11 × 9 = 2
Lescher et al. 2016 [27]	Germany	2016	Retrospective	22	16	23	60.63	9.6	Aneurysm recurrence	15 months	10 × 8 = 1, 9 × 7 = 1
Liebig et al. 2015 [28]	Germany	2015	Retrospective	47	NA	52	NA	NA	Residual = 1, recurrence = 3,	Mean = 4 months	NA
Lubicz et al. 2014 [29]	Multi-institution	2014	Retrospective	45	34	45	56.3	9.6	Residual aneurysm or neck	Mean = 7.5 months	NA
Mouchtouris et al. 2022 [30]	USA	2022	Retrospective	110	79	115	62.7	12.2	Asymptomatic reperfusion = 2, neck reperfusion and symptomatic acute thrombotic occlusion of the anterior cerebral artery = 1,	Mean = 11 months	NA
Ozpeynirci et al. 2019 [31]	Germany	2019	Retrospective	45	33	47	60	NA	Aneurysm recanalization = 1, initially partially occluded = 1	6 months	NA
Pierot et al. 2012 [32]	France	2012	Prospective	20	16	21	58.75	10.15	Aneurysm recanalization	8 months	NA
Pierot et al. 2016 [33]	France	2016	Prospective	62	39	63	56.6	9.8	Aneurysm remnant = 4 (2 aneurysms growing between two imaging controls)	Mean = 27 months	NA
Pierot et al. 2019 [34]	France	2019	Prospective	130	NA	130	NA	NA	Aneurysm remnant	5 years	7 × 5 = 1, 7 × 3 = 1, 7 × 4 = 1, 4 × 3 = 1
Pierot et al. 2023 [6]	France	2023	Prospective	106	68	106	NA	NA	Residual aneurysm or neck	Mean = 7.5 months	NA
Semeraro et al. 2023 [35]	Italy	2023	Retrospective	104	58	104	58.6	11.8	NA	NA	NA
Spelle et al. 2023 [4]	France	2021	Prospective	60	31	60	54.5	11.5	Aneurysm remnant or recurrence	12 months	NA
Srinivasan et al. 2022 [7]	USA	2022	Retrospective	30	20	30	57	12.7	Aneurysm residual or growth = 12, neck residual = 12 cases, compaction or deformation of WEB = 6, aneurysm rebleed = 1, new aneurysm growth and rupture = 1 was noted in 6 cases	Mean = 13.5 months	7 × 3 = 4, 8 × 5 = 1, 7 × 4 = 2, 9 × 4 = 3, 8 × 3 = 3, 8 × 4 = 2, 10 × 5 = 1, 4 × 3 = 1, 9 × 6 = 2, 6 × 4 = 1, 6 × 3 = 1, 4 × 5 = 1, 4 × 7 = 1, 8 × 5 = 1, 11 × 6 = 1, 7 × 5 = 1, 10 × 6 = 1, 8 × 5 = 1, 4 = 1
Youssef et al. 2021 [36]	USA	2020	Retrospective	48	32	48	57.8	15.2	Aneurysm recurrence	4 months	NA

**Table 2:** Baseline characteristics of the included studies

Parameter	Value
Female sex	1019/1501 (67.9%)
Mean age, yrs	52.3 (2.8)
Lost to follow-up	323/1489 (21.7%)
Past medical history	
Dyslipidemia	21/115 (18.3%)
Diabetes	4/48 (8.3%)
Hypertension	135/236 (57.2%)
Smoker	138/275 (50.2%)
Antiplatelet	27/109 (24.8%)
Initially treated ruptured status	
Ruptured	536/1651 (32.5%)
Unruptured	1115/1651 (67.5%)
Retreatment ruptured status	
Ruptured	7/135 (5.2%)
Unruptured	128/135 (94.8%)
Initially treated aneurysm location	
MCA	477/1444 (33%)
ICA	299/1141 (26.2%)
PCA	73/356 (20.5%)
ACA	142/844 (16.8%)
AcomA	469/1672 (28%)
BA	364/1520 (21.8%)
PcomA	102/1017 (10%)
PICA	20/682 (2.9%)
VA	29/571 (5%)
SCA	9/480 (1.9%)
Retreatment aneurysm location	
MCA	40/110 (36%)
ICA	5/41 (12%)
PCA	1/8 (12%)
AcomA	39/129 (30%)
BA	38/118 (32%)
PcomA	8/56 (14%)
PICA	1/7 (14%)
Aneurysm size	
Mean aneurysm height, mm	6.9 (1.05)
Mean neck width, mm	4. (0.7)
Dome to neck ratio (range)	(1.3–1.7)
Initially treated WEB types	
WEB SL	852/1334 (63.9%)
WEB SLS	129/1011 (12.8%)
WEB DL	373/1214 (30.7%)

*MCA* middle cerebral artery; *ICA* internal carotid artery; *ACA* anterior cerebral artery; *PCA* posterior cerebral artery; *AcomA* anterior communicating artery; *PcomA* posterior communicating artery; *BA* basilar artery; *PICA* posterior inferior cerebellar artery; *SCA* superior cerebellar artery;

### Initial treatment occlusion and aneurysm location

**Fig. 2:**
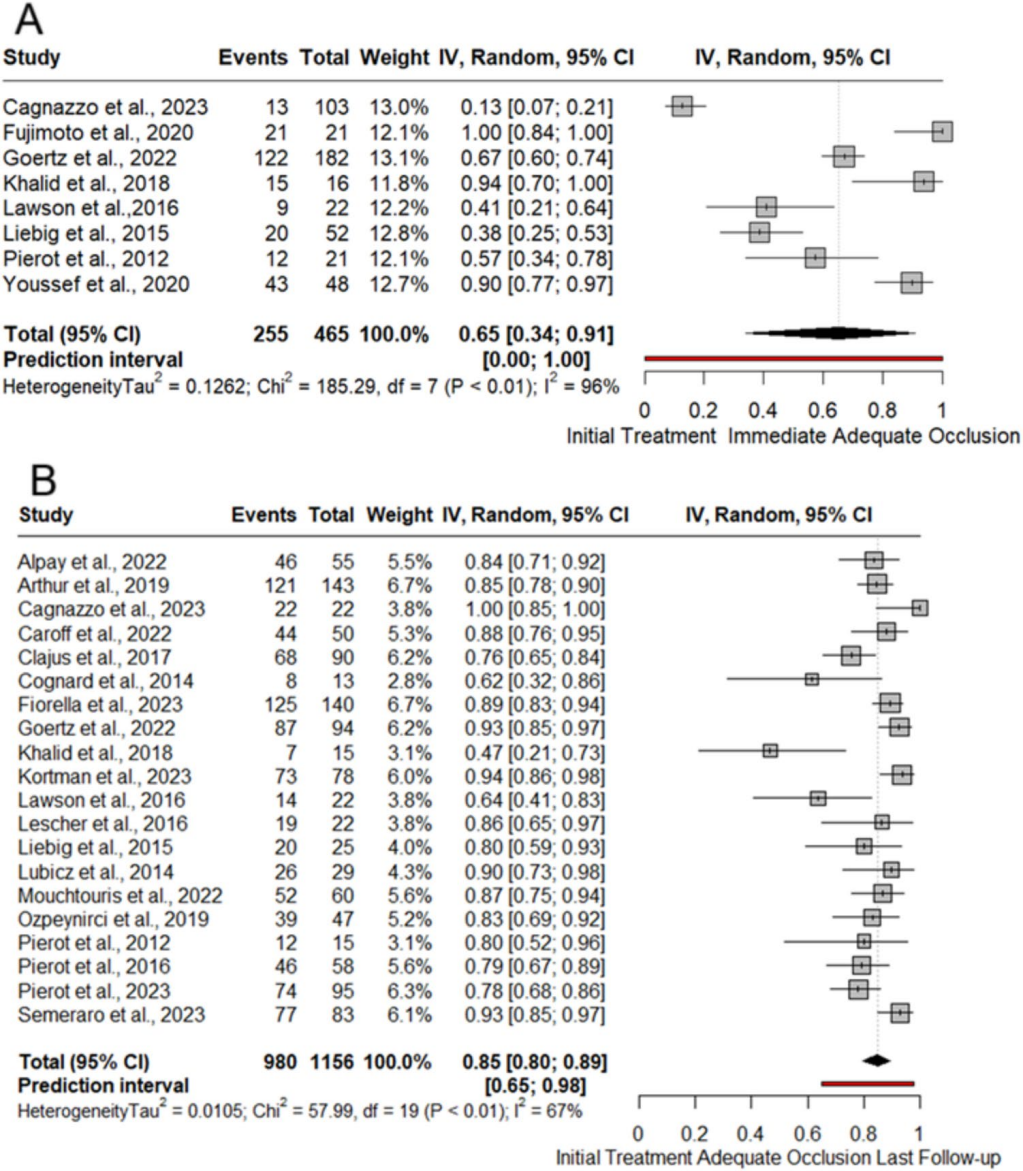
Meta-analysis forest plot for initial treatment adequate occlusion post-intervention (**A**) and last follow-up (**B**)

Immediate post-operation adequate occlusion rate in patients initially treated with WEB among 465 patients were reported in nine studies [12, 23, 25, 26, 28, 32, 36, 38]. The rate of post-operative adequate occlusion was 65% (8 studies; 255/465; 95%CI, 34%− 90%) Fig. 2A. Twenty studies [3, 12, 13, 15, 18–22, 25–33, 35, 38] with 1156 patients reported adequate occlusion at last follow-up. Rate of adequate occlusion at the last follow-up was 84.7% (20 studies, 980/1156, 95%CI: 79%− 89%) Fig. 2B.

The most common sites of aneurysms in initial treatment were middle cerebral artery (24 studies, 36%, 95%CI: 28%− 44%), anterior communicating artery (24 studies, 30%, 95%CI: 25%− 44%), basilar artery (24 studies, 21%, 95%CI: 15%− 28%), and internal cerebral artery (24 studies, 5%, 95%CI: 3%− 7%), which difference was significant (*P*-value < 0.0001).

Meta-regression was conducted to evaluate heterogeneity observed in analyses of initial treatment adequate occlusion at last follow-up (Table 3). The heterogeneity observed in adequate occlusion rate was explained by the aneurysms located at middle cerebral artery, internal carotid artery, posterior communicating artery, and higher aneurysm height and neck width (Table 3).

**Table 3:** Meta-regression for outcomes with significant heterogeneity

	Initial Treatment Adequate Occlusion at Follow-up			Retreatment Rate		
Covariate	Coefficient	*p*-value	R^2	Coefficient	*p*-value	R^2
Year of publication	0.0002	0.97	0.0%	− 0.0004	0.95	0.0%
Sample Size	− 0.0004	0.24	0.0%	− 0.0003	0.4	0.0%
Female sex	− 0.0008	0.23	6%	− 0.0006	0.43	0.0%
Aneurysms number	− 0.0004	0.27	0.0%	− 0.0004	0.43	0.0%
Age	0.0007	0.95	0.0%	− 0.0019	0.86	0.0%
Ruptured	− 0.0008	0.34	4.2%	− 0.0008	0.36	1.3%
MCA	− 0.0116	**0.0147**	25%	0.0005	0.78	0.0%
ICA	− 0.0028	0.058	21%	− 0.0002	0.4	0.0%
AcomA	0.0241	**0.0055**	15%	− 0.0017	0.31	0.0%
BA	− 0.0005	0.71	0.0%	− 0.0001	0.96	0.0%
PcomA	− 0.0189	**0.0212**	92%	− 0.0085	0.057	98.12%
Partially thrombosed	NA	NA	NA	0.0092	0.14	0.0%
Aneurysm height	− 0.0489	**0.0047**	53%	0.0488	**0.0033**	89.10%
Neck width	− 0.0692	**0.03**	51%	0.0739	**0.032**	73.85%
Dome to neck	− 0.1760	0.57	0.0%	− 0.1615	0.66	0.0%
WEB SL	− 0.0004	0.45	0.0%	− 0.0003	0.65	0.0%
WEB SLS	− 0.0039	0.28	0.0%	− 0.003	0.41	0.0%
WEB DL	− 0.0003	0.81	0.0%	− 0.00001	0.99	0.0%
WEB size (Diameter)	NA	NA	NA	0.0076	0.82	0.0%
WEB size (Height)	NA	NA	NA	− 0.0042	0.88	0.0%

*MCA* middle cerebral artery; *ICA* internal carotid artery; *AcomA* anterior communicating artery; *PcomA* posterior communicating artery; *BA* basilar artery

Aneurysm located at anterior communicating artery, and lower aneurysm height and neck width were associated with significantly higher and aneurysms located at middle cerebral artery and posterior communicating artery was related to lower initial-treatment adequate occlusion at last follow-up. Other covariates did not show a significant association with the initial-treatment occlusion rate at last follow-up, while some were not evaluated given the lack of power (less than 7 studies).

### Retreatment rate and location

**Fig. 3:**
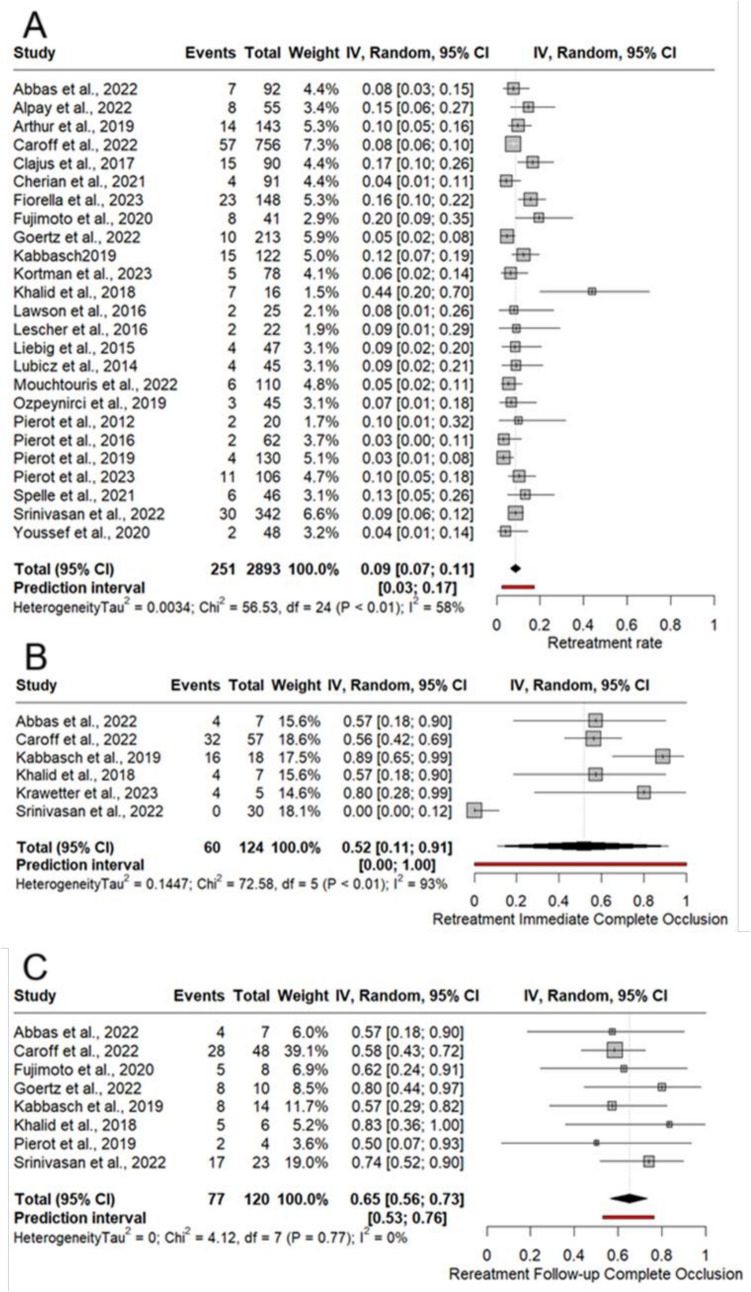
Meta-analysis forest plot for retreatment rate (**A**), retreatment complete occlusion post-operative (**B**), and retreatment complete occlusion at last follow-up (**C**)

Retreatment rate was reported in 24 studies [3, 4, 7, 10, 12, 13, 15, 18–21, 23–34, 36] among 2802 patients. The overall retreatment rate was 8.6% (24 studies, 251/2893, 95%CI: 6.5%− 10.9%) Fig. 3A. Meta-regression illustrated that the percentage of aneurysm height, neck width, and posterior communicating artery are the sources of heterogeneity for retreatment rate (Table 3). Aneurysm height and neck width significantly increased the calculated effect for retreatment, favoring a lower retreatment rate in lower aneurysm height and neck width (Table 3).

There was no significant difference between ruptured (6 studies, 0.35%, 95%CI: 0%− 7.3%) and unruptured (6 studies, 0.36%, 95%CI: 0.43%− 16.8%) aneurysms in retreatment rate (*P*-value = 0.64) [3, 21, 26, 27, 31, 32]. The retreated aneurysm location was reported for 137 patients. The middle cerebral artery was the most common location for retreatment (14 studies, 35%, 95%CI: 25%− 45%) followed by basial artery (14 studies, 31.1%, 95%CI, 25.4%− 37.1%). Subgroup analysis showed a significant difference (*P*-value = 0.0002) between the aneurysm retreatment rate location Supplement Fig. 1.

### Retreatment occlusion rate

Immediate complete occlusion after the retreatment was reported by six studies [7, 9, 10, 20, 24, 25] with a total of 124 cases. The immediate complete occlusion rate post retreatment was 52% (six studies, 95%CI, 11%− 91%) Fig. 3B. The immediate adequate occlusion (not including complete occlusions, RROC = 2) following the aneurysmal retreatment was 34% (six studies, 46/124, 95%CI: 11%− 91%) Supplement Fig. 2. At the last follow-up, the complete occlusion rate (Fig. 3C) was and adequate occlusion rate were 65% (8 studies, 77/120, 95%CI: 56%− 73%) and 96.7% (8 studies, 112/120, 95%CI: 91%− 100%), respectively Supplement Fig. 3.

Subgroup analysis showed that stent-assisted coiling achieved a 100% (95%CI: 57%− 100%) (*P*-value < 0.01) adequate occlusion rate post-operatively, while coiling demonstrated 67% (95%CI: 0%− 100%) adequate occlusion rate Supplement Fig. 4. At last follow-up, stent-assisted coiling (74%, 95%CI: 16%− 100%) and microsurgical clipping (65%, 95%CI: 31%− 94%) showed highest rate of complete occlusion (*P*-value = 0.63) Supplement Fig. 5. The rate of adequate occlusion (not including complete occlusions, RROC = 2) showed no significant difference (*P*-value = 0.14), although the direction of the association suggested higher odds of adequate occlusion in patients receiving flow diverter (58%, 95%CI: 21%− 91%) compared to other modalities Supplement Fig. 6.

### Retreatment approaches

Twenty-six studies [3, 4, 7, 9, 10, 12, 13, 15, 18–21, 23–25, 27–34, 36, 38] with 257 aneurysms reported the retreatment approach following WEB placement. Stent-assisted coiling (32.3%, 83/257), microsurgical clipping (12.1%, 31/257), coiling (15.6%, 40 out of 257), and flow diversion (27.2%, 70/257) were utilized as retreatment methods. WEB (7.4%, 19/257), flow redirection endoluminal device (FRED) (1.2%, 3/257), and Pipeline (3.1%, 8/257) were used as flow diverters.

### Recurrence-related to WEB type

Seventeen studies [3, 7, 9, 10, 15, 18, 20, 21, 23–29, 33, 34] reported types of WEB that led to aneurysm recurrence among 370 patients. WEB SL (17 studies, 81%, 95%CI: 58%− 97%) showed highest proportion, while WEB SLS (17 studies, 14%, 95%CI: 3%− 29%) illustrated the lowest recurrence rate Supplement Fig. 7. The test for subgroup analysis was significant (*P*-value < 0.01).

### Complications

After the initial treatment by WEB, 393 out of 1323 patients experienced (29.7%) complications, of which the procedure-related mortality rate was 7.5% (44/588) Table 4. Overall complication following retreatment was 10.5% (12/114) Table 4. Additionally, microsurgical clipping (11.8%, 4/34) showed highest overall complication rate of all retreatment methods. Furthermore, detailed specific WEB-related complications were device protrusion (3.7%, 17/462), thromboembolic (3.3%, 10/299), extravasation (1.9%, 2/108), aneurysm perforation (0.9% 2/227) leading to SAH, dissection (1.7%, 2/116), rebleeding (1.9%, 2/108), premature detachment of the device (3.1%, 2/65), device malpositioning (2.1%, 1/48), overestimation of WEB size (0.9%, 1/110), WEB stuck in the microcatheter (1.6%, 1/62), and unable to deploy WEB device (4.1%, 2/48).

**Table 4:** Reported complications of initial treatment and retreatment

	Initially Treated Complications		Retreatment Complications based on Approach
Intraoperative aneurysm rupture	27/756 (3.6%)	Stent-assistant coiling	3/84 (3.6%)
Thromboembolic events	102/1124 (9.1%)	Clipping	4/34 (11.8%)
Delayed adverse events	95/854 (11.1%)	Coiling	3/46 (6.5%)
Procedure-related morbidity	120/726 (16%)	WEB	1/21 (4.8%)
Procedure-related Mortality	44/588 (7.5%)	Flow diverter	3/73 (4.1%)
Overall rate	393/1323 (29.7%)	Overall rate	12/114 (10.5%)

#### Quality assessment

The risk of bias was low in 16, moderate in 7, and high in 6 studies with no included studies had critical risk of bias Supplement Figs. 8 and 9.

Furthermore, contour-enhanced funnel plot for assessment of publication bias showed the low publication bias in outcomes of interest Supplement Figs. 10 and 11.

## Discussion

### Summary of findings

Our systemic review and meta-analysis included 29 clinical studies investigating recurrence and retreatment after successful WEB placement in patients with intracranial aneurysms. Our findings showed that about one out of ten aneurysms required retreatment. In addition, stent-assisted coiling significantly demonstrated highest efficacy in achieving adequate occlusion at post-operation. However, the evidence was not enough to significantly showed the superior retreatment modality at the last follow-up. On the other hand, adequate occlusion rate after initial treatment and retreatment were 82% and 97% at last follow-up, respectively.

### Retreatment and causes of failure after WEB

Our pooled findings showed a 9% retreatment rate. The retreatment rate across the included studies ranged from 3.1% [34] to 46.7% [25]. This appears slightly higher than retreatment rates associated with microsurgical clipping (4%) [39], and stent-assisted coiling and flow diverter (5%) [40], but lower than coiling (10%) [41, 42]. Additionally, we found that larger aneurysm height and neck width were significantly associated with a higher retreatment rate. It is well established that larger aneurysms have a higher recurrence rate and an increased need for retreatment [7, 43]. Furthermore, the largest available WEB device, measuring 11 mm in width and 9–10 mm in height, poses significant challenges in selecting the optimal size for treating large aneurysms.

WEBCAST [44] and WEB-IT [19] trials demonstrated very high safety and efficacy of the WEB device, while postmarket studies have shown considerably lower adequate and complete occlusion rates, indicating a strong dependence on patient and aneurysm selection. The application of multiple devices in a single session was observed in 20% of cases, and the challenge of selecting optimal size of WEB device was noted, even in high-volume, experienced centers [37, 45]. Suboptimal case selection, contributing to higher inadequate and treatment failure rates, remains a prevalent issue in post-market and early-adopter studies [37].

Our meta-analysis encompassed the use of the WEB device in less common sidewall aneurysms, such as those in the posterior inferior cerebellar artery (4.1%) and posterior communicating artery (10.3%). On the other hand, we showed that WEB-SL can carry a higher risk of recurrence than WEB-SLS and WEB-DL. Higher usage of WEB-SL and institution-specific factors may affect the incidence of retreatment [23].

### Retreatment approaches

Our findings showed that post-retreatment complete occlusion rate was 65% and the adequate occlusion rate was 96.7% at last follow-up. We found no significant difference between retreatment strategies in long-term occlusion rate. However, the stent-assisted coiling showed a significantly higher adequate occlusion immediate post-intervention.

Microsurgical clipping remains gold standard for treating cerebral aneurysms, providing a definitive option for permanent aneurysm occlusion, as recurrence is exceedingly rare [7, 10, 34, 46]. Although several large studies have identified challenges with clipping after coiling—such as potential for parent artery occlusion due to displaced coils and scarring surrounding extruded coils—findings regarding microsurgical treatment following WEB device failure have been less consistent. Studies [7, 10, 34] utilizing microsurgery after WEB recurrence demonstrated that the WEB device remains intrasaccular, and its soft, compressible nature allows for clip placement without requiring device extraction, unlike the microsurgery of previously coiled aneurysms [47].

Majority of cases in this study were retreated using endovascular techniques. Several factors, including aneurysm location, patient preference for less invasive approaches, and institution-specific considerations, influenced decision. Studies have demonstrated that endovascular treatment is a safe, effective, and technically straightforward with excellent angiographic outcomes and limited complications [10, 24, 44, 48]. Although our findings showed that long-term radiological outcomes were comparable between two approaches, we observed a lower complication rate with endovascular treatment compared to clipping.

Studies evaluated radiographic outcomes using WOS and RROC. This has sparked debate and confusion, particularly because the clinical implications of a neck remnant in the context of WEB embolization are not as well-defined as they are for coil embolization [49]. In this setting,"adequate"occlusion indicates that the aneurysm is stable, unlikely to rupture, and requires no more treatment. In a study [7], eleven neck remnants were deemed at risk and deserving of retreatment. Additionally, progression of RROC or WOS scores can occur during long-term follow-up in aneurysms initially deemed adequately occluded. This highlights that neck remnants following WEB embolization may necessitate close follow-up, as they may not provide same long-term stability observed with coiled aneurysms [49].

### Strengths, limitations, and future directions

The current study is the largest meta-analysis to comprehensively evaluate the retreatment strategy after the WEB treatment with a large and diverse sample which allows for adequately powered analysis of real-world data. In addition, we did a meta-regression to combine and compare findings from multiple clinical studies while adjusting for the effects of available covariates on retreatment rate and initial treatment adequate occlusion rate.

Some limitations should be acknowledged. Firstly, the available literature on retreatment strategy for aneurysm recurrence after the WEB treatment was limited, and some studies lacked the necessary data to conduct a meta-analysis. Additionally, there was considerable heterogeneity among included studies, possibly because of different institution- and operator-specific retreatment indications, various follow-up periods, and a wide range of retreatment options. Although meta-regression identifies the source of the heterogeneity in retreatment rate and initial occlusion status, regression was not applicable for retreatment occlusion status, retreatment location, and retreatment strategies because of a lack of statistical power due to limited reports [14]. Furthermore, no studies provided detailed information on variability in aneurysm/branch configurations, so we cannot consider these factors in analysis. Future studies can address these gaps. However, we reported the indications and reasons for aneurysm retreatment by each study after initial WEB embolization; retreatments are generally conducted in the absence of established criteria. For the retreatment, most studies did not report the complications in detail and reported them as a rate based on each technique, so we were unable to document complications same way we documented the initial treatment.

Future studies can focus on more specific patient selection based on each retreatment strategy, considering the complex anatomical variation and evaluating retreatment's cost-effectiveness and long-term outcomes. Studies optimizing retreatment strategies by defining the exact criteria for each treatment method and directly comparing each endovascular approach and surgical treatment may be a valuable addition to the literature.

## Conclusions

The current systematic review and meta-analysis suggest that roughly 9% of aneurysms treated with WEB required retreatment. The overall complete and adequate retreatment occlusion was high and although the stent-assisted coiling showed a higher adequate occlusion rate post-operatively, there was no significant difference between retreatment strategies in long-term radiological outcomes.

## Supplementary Information

Below is the link to the electronic supplementary material.

Supplementary file1 (DOCX 423 KB)

## Data Availability

No datasets were generated or analysed during the current study.
