# Supplementary material for: Retreatment rate and strategies for recurrent and residual aneurysms after Woven EndoBridge (WEB) treatment: a comprehensive systematic review and meta-analysis
Source: Neurosurg Rev. 2025 May 2;48(1):400. doi: 10.1007/s10143-025-03532-y (PMC12048415; doi:10.1007/s10143-025-03532-y)
Supplement: Supplementary file 1 — Supplementary file1 (DOCX 423 KB) [file 10143_2025_3532_MOESM1_ESM.docx]

**eAppendix**

**Retreatment Strategy for Aneurysm Recurrence After the Woven EndoBridge (WEB) Treatment: A Comprehensive Systematic Review and Meta-analysis With a Case Illustration**

**Supplementary SFigures Legend:**

**SFigure 1.** Meta-analysis forest plot for retreated aneurysm location

**SFigure 2.** Meta-analysis forest plot for immediate adequate occlusion

**SFigure 3.** Meta-analysis forest plot for last follow-up adequate occlusion

**SFigure 4.** Forest plot for subgroup analysis of adequate occlusion rate post-operatively

**SFigure 5.** Forest plot for subgroup analysis of complete occlusion rate at last follow-up

**SFigure 6.** Forest plot for subgroup analysis of adequate occlusion rate at last follow-up

**SFigure 7.** Forest plot for subgroup analysis of types of WEB that led to aneurysm recurrence

**SFigure 8**. Traffic light plot showing the risk of bias of the included studies

**SFigure 9**. Bar plot showing the risk of bias of the included studies

**SFigure 10**. Contour-enhanced funnel plot for retreatment rate showing the risk of publication bias of the included studies

**SFigure 11**. Contour-enhanced funnel plot for initial treatment adequate occlusion at last follow-up showing the risk of publication bias of the included studies


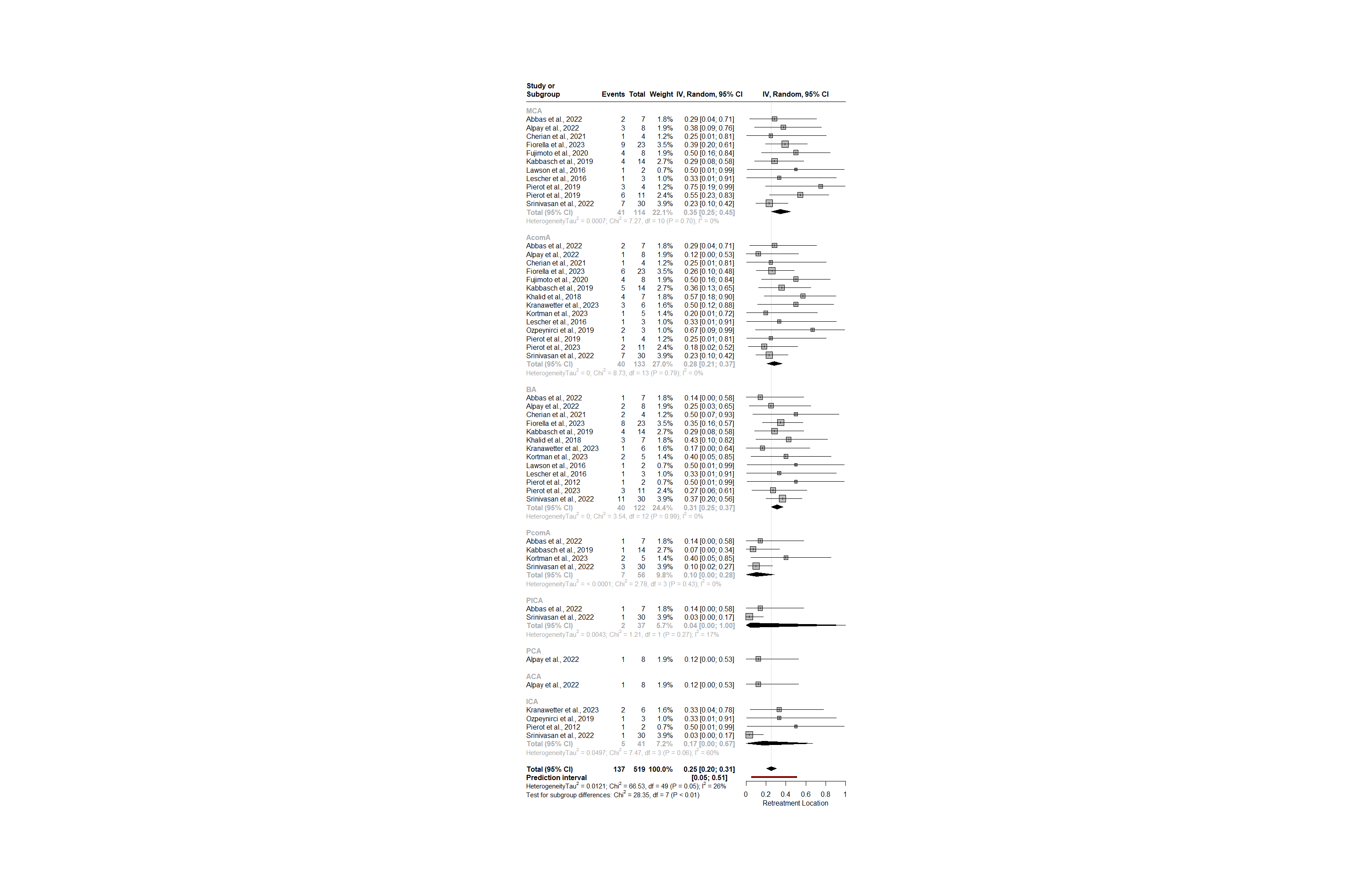


**SFigure 1.** Meta-analysis forest plot for retreated aneurysm location


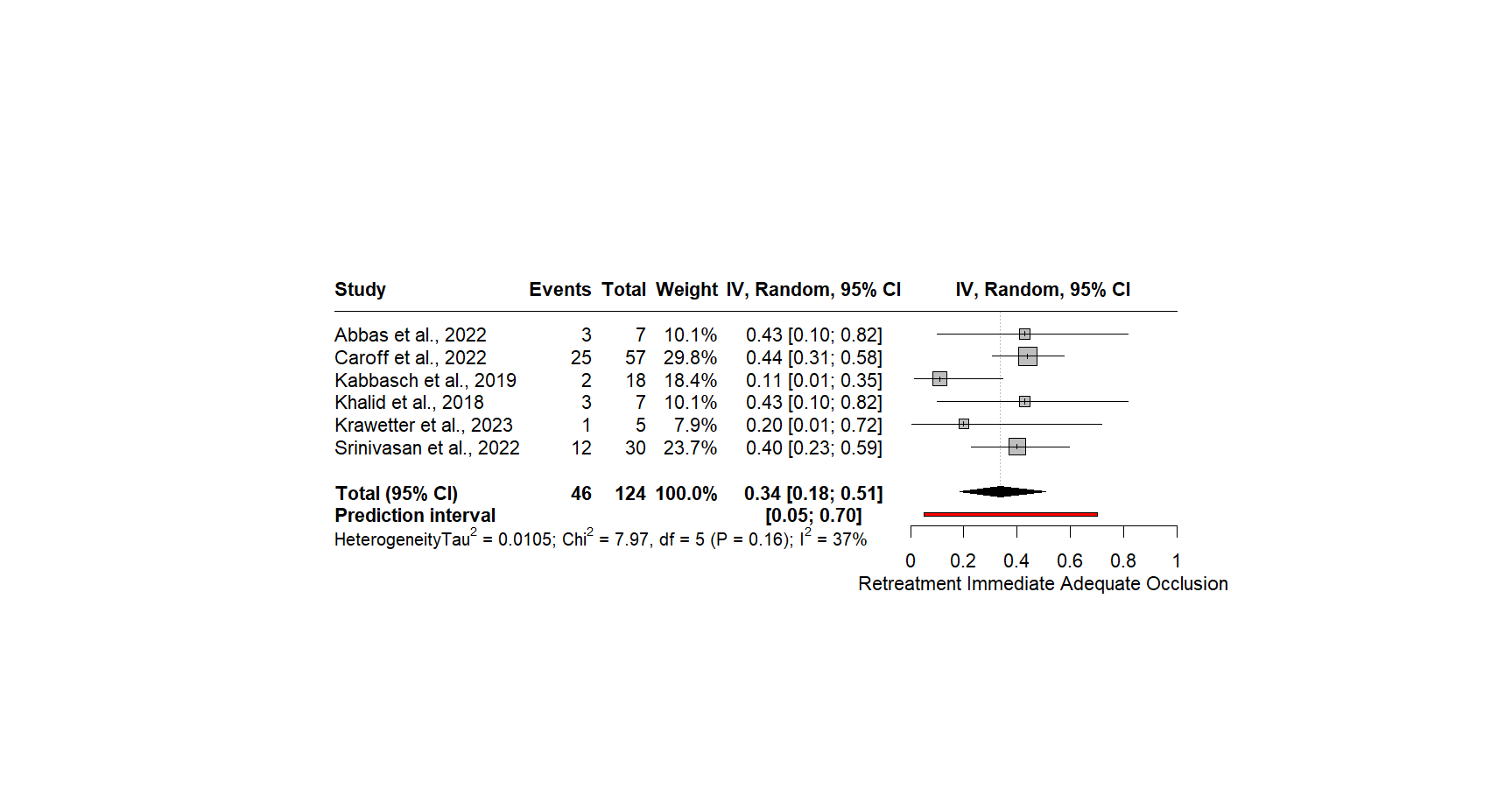


**SFigure 2.** Meta-analysis forest plot for immediate adequate occlusion


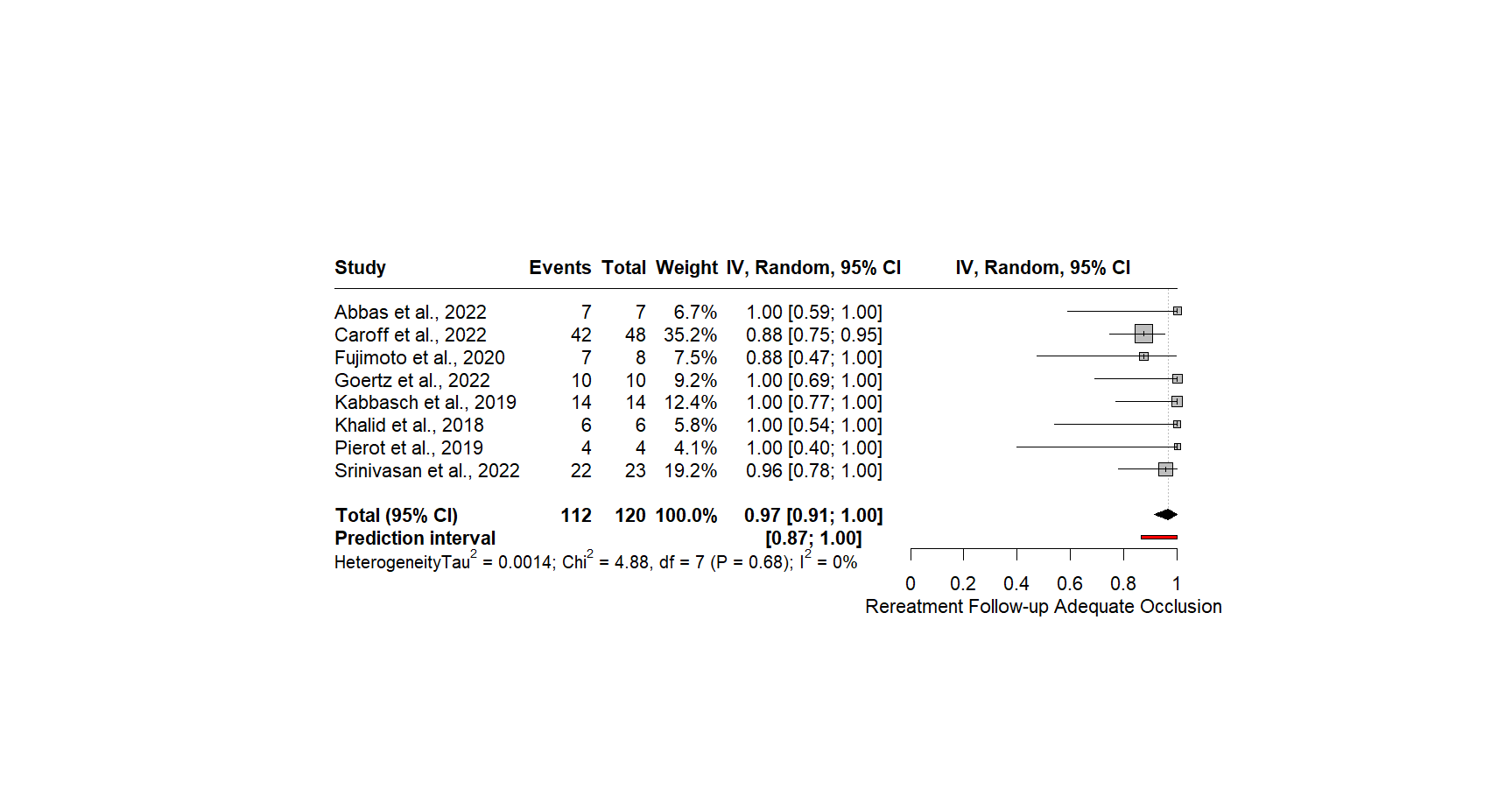


**SFigure 3.** Meta-analysis forest plot for last follow-up adequate occlusion


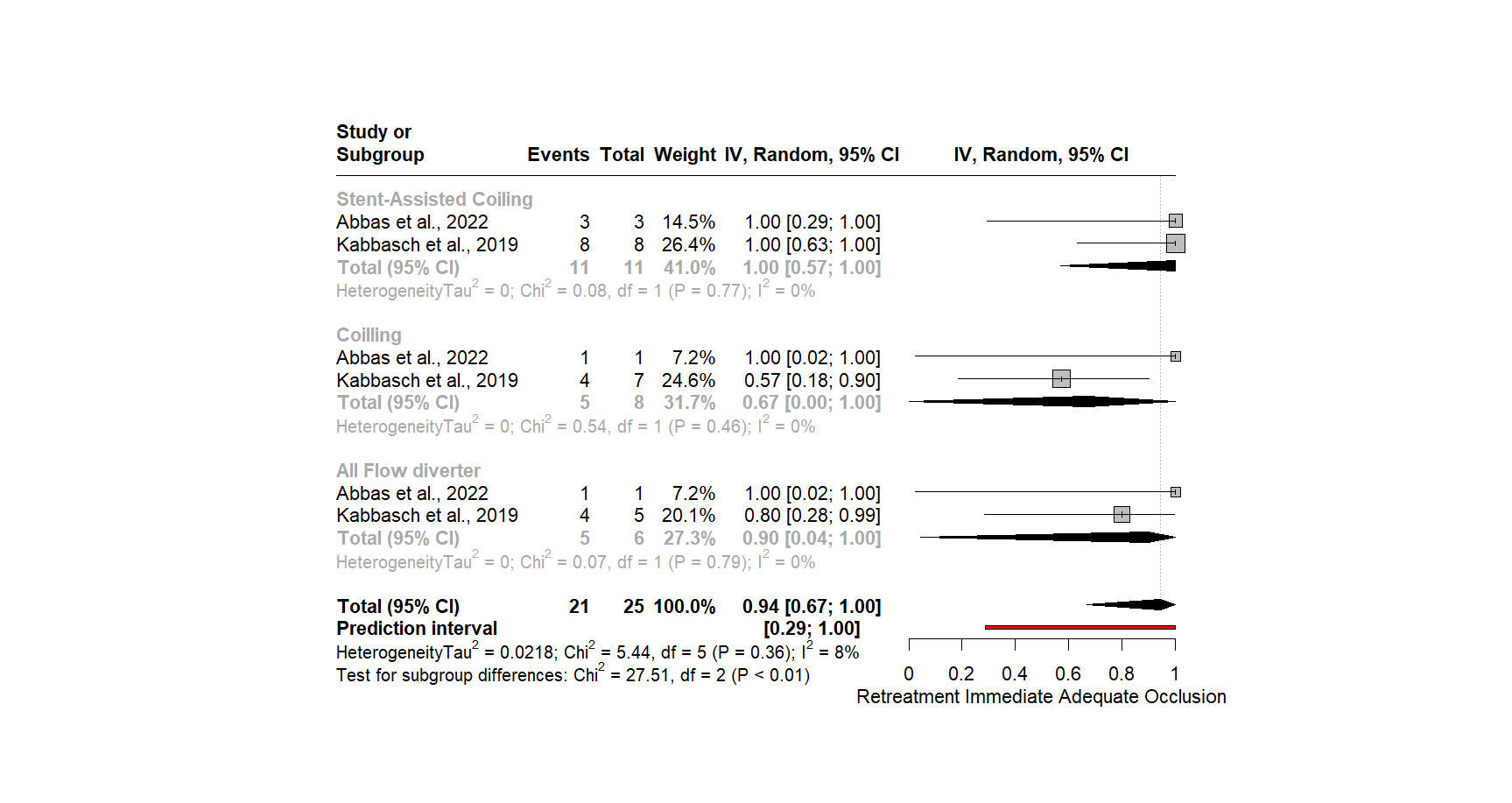


**SFigure 4.** Forest plot for subgroup analysis of adequate occlusion rate post-operatively


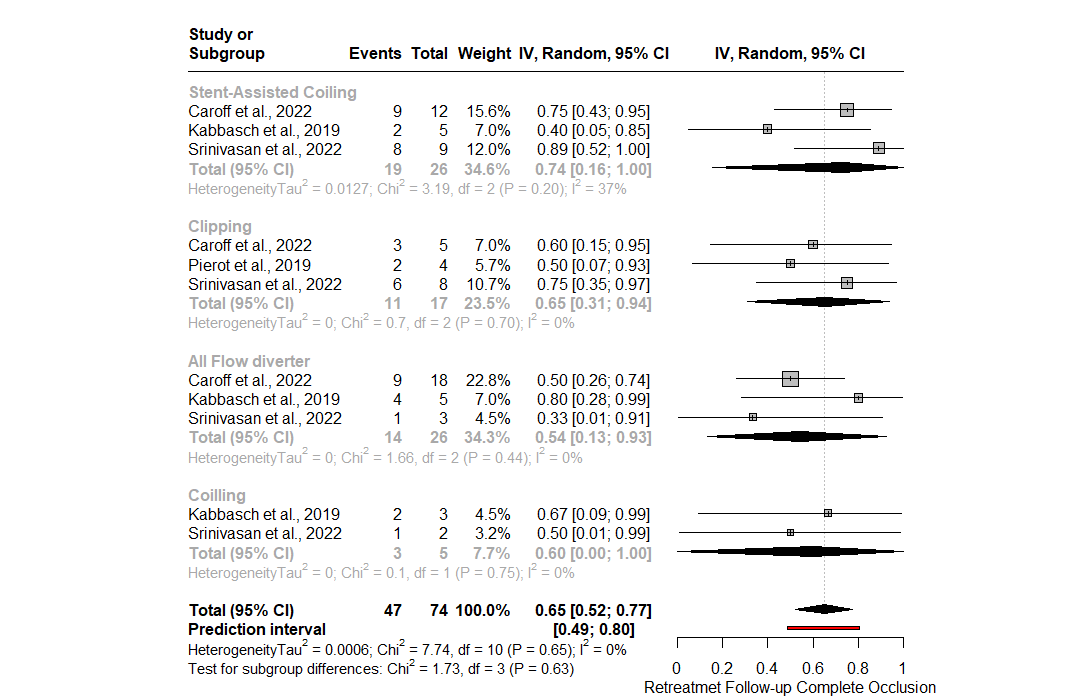


**SFigure 5.** Forest plot for subgroup analysis of complete occlusion rate at last follow-up


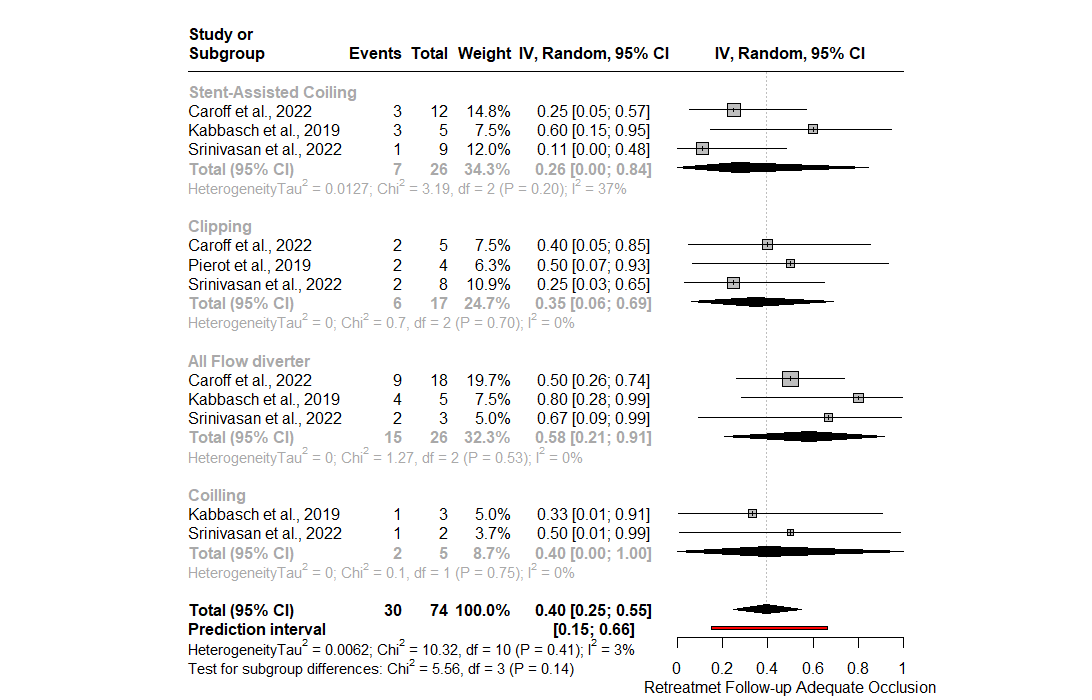


**SFigure 6.** Forest plot for subgroup analysis of adequate occlusion rate at last follow-up


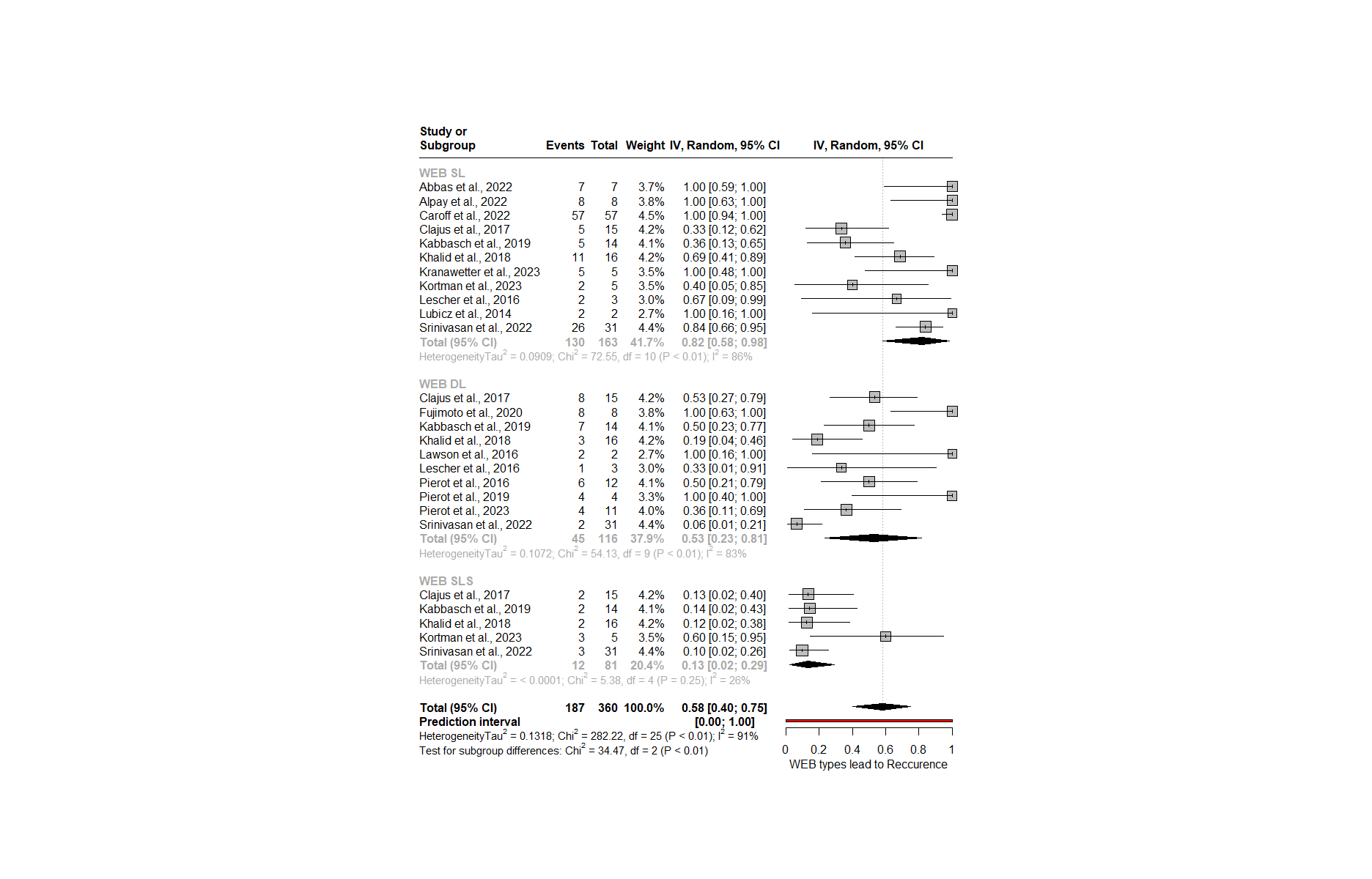


**SFigure 7.** Forest plot for subgroup analysis of types of WEB that led to aneurysm recurrence


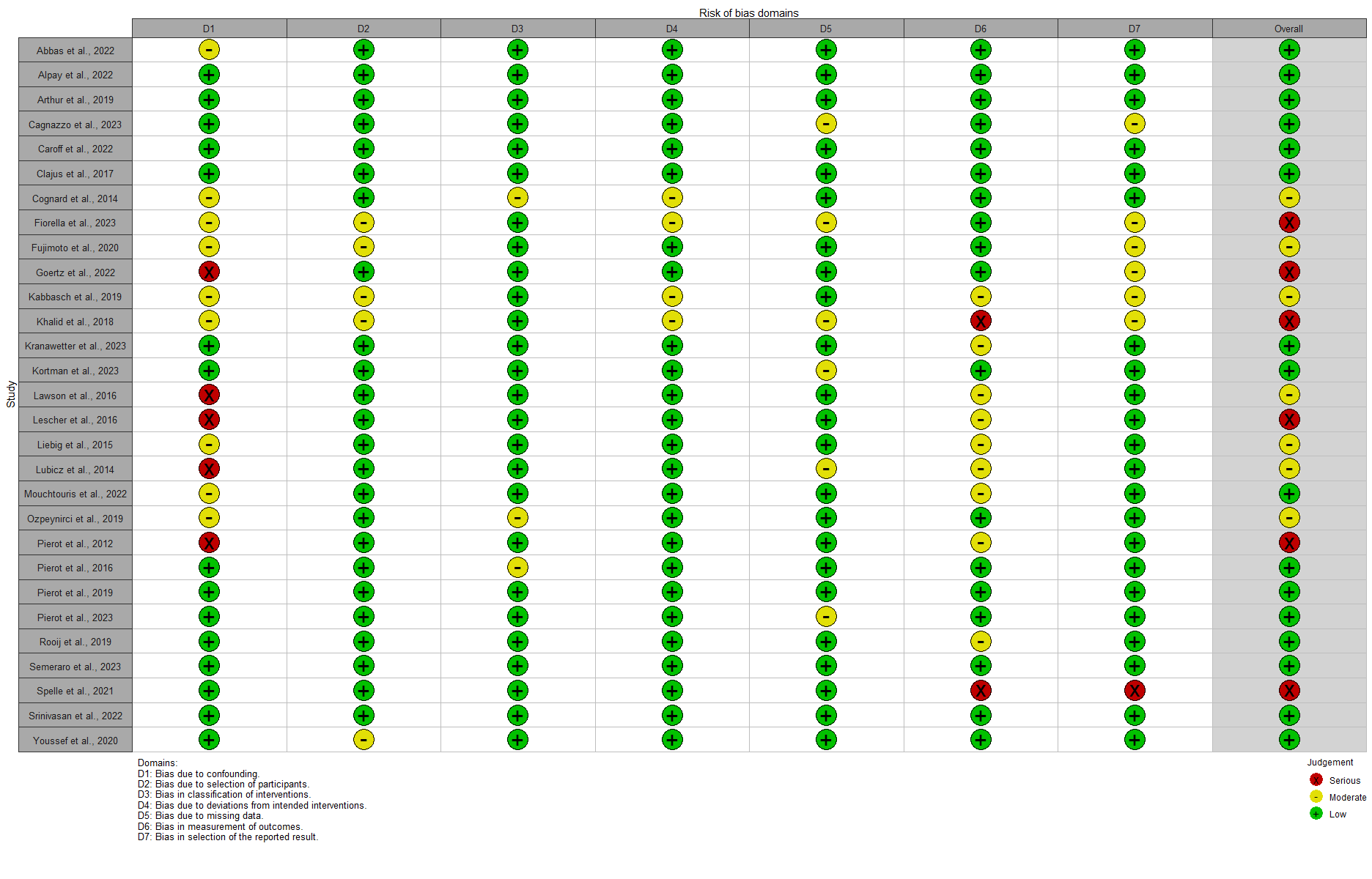
 **SFigure 8**. Traffic light plot showing the risk of bias of the included studies


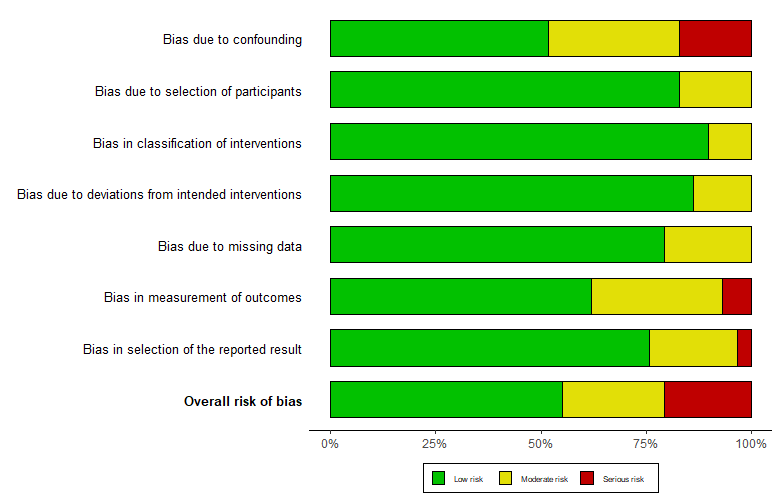


**SFigure 9**. Bar plot showing the risk of bias of the included studies


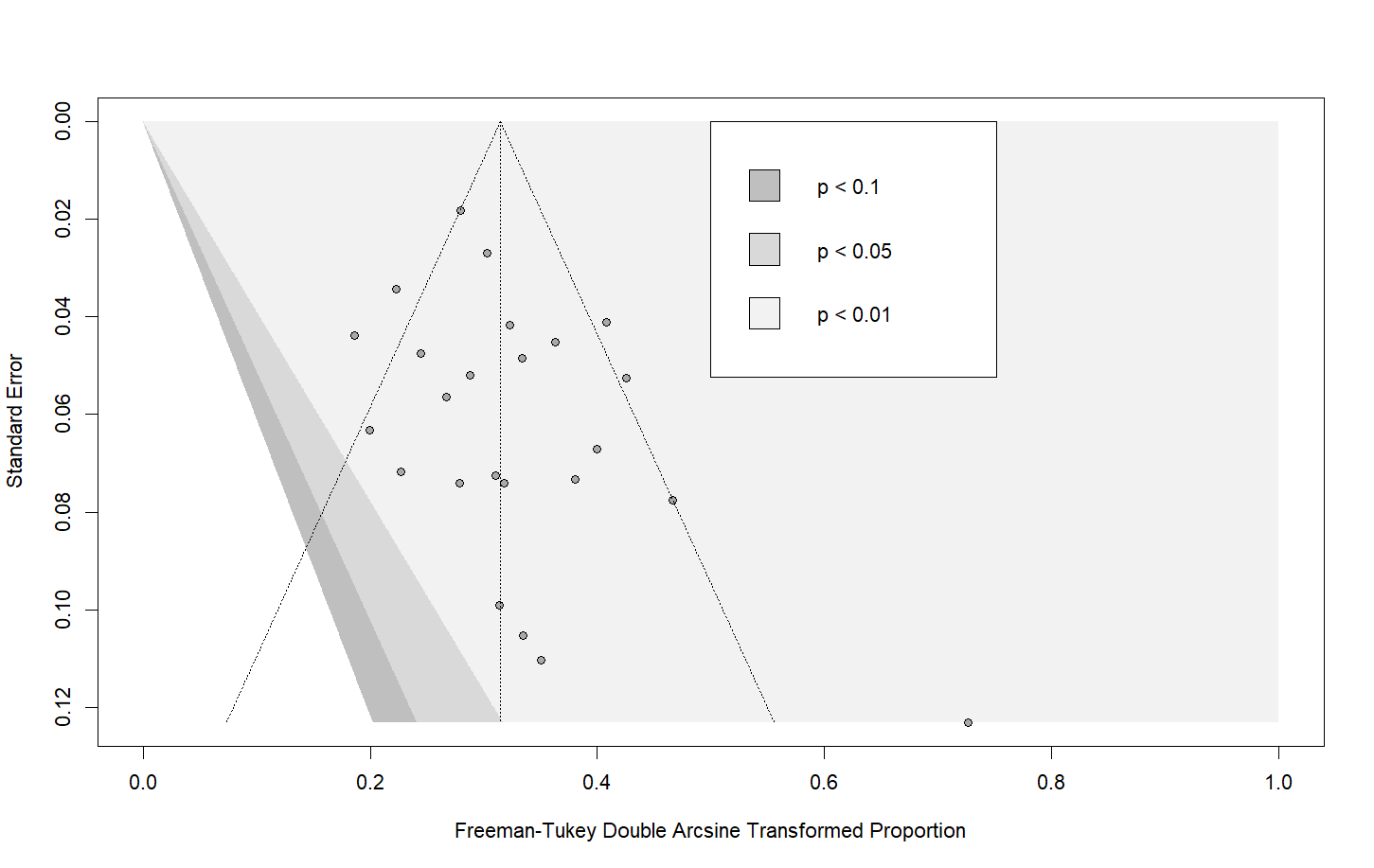


**SFigure 10**. Contour-enhanced funnel plot for retreatment rate showing the risk of publication bias of the included studies

**
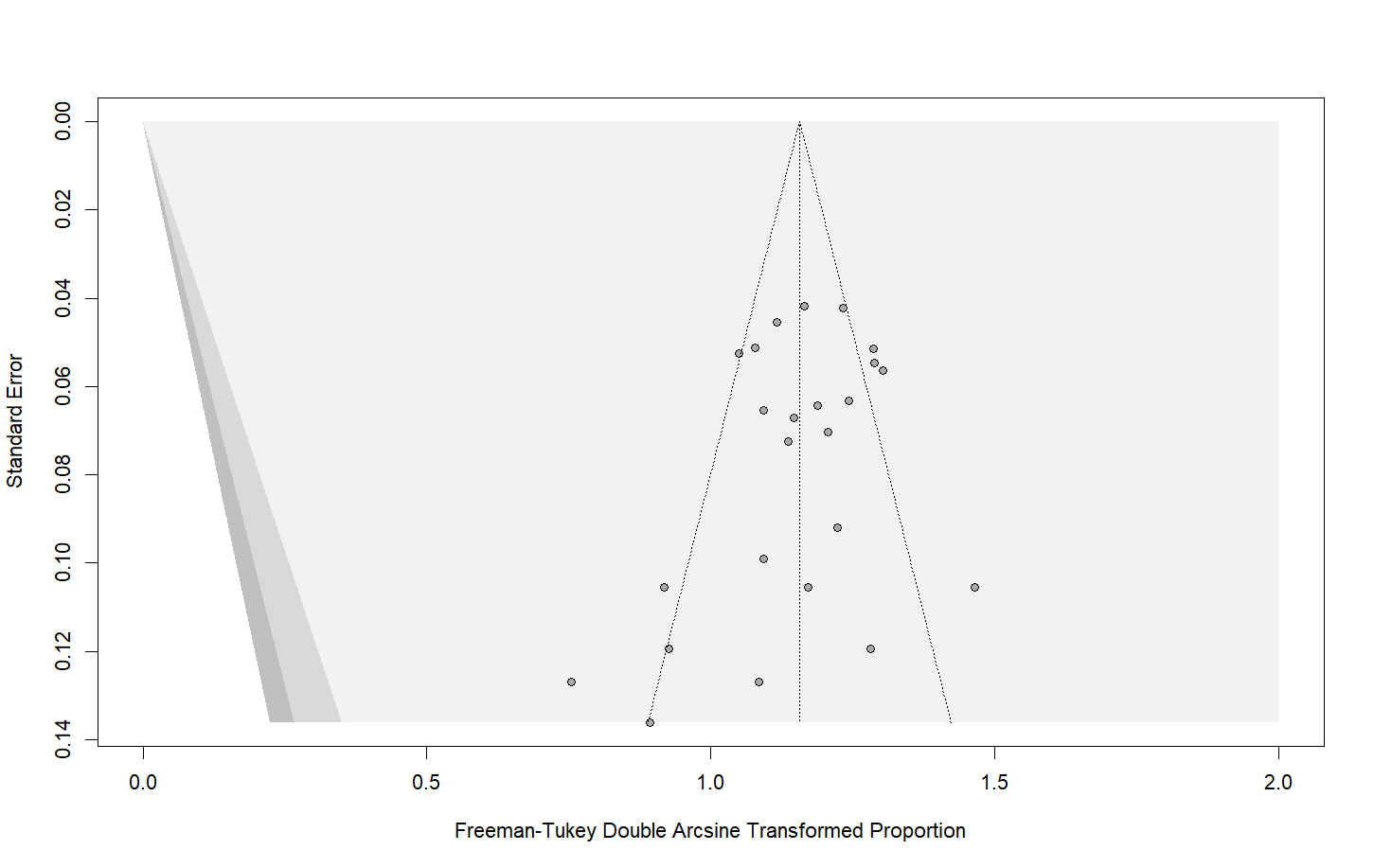
**

**SFigure 11**. Contour-enhanced funnel plot for initial treatment adequate occlusion at last follow-up showing the risk of publication bias of the included studies

**STable 1.** Search strategy for selection of the studies

| Search | Database | Query | Date | Results | Duplicate | Excluded | Included |
| --- | --- | --- | --- | --- | --- | --- | --- |
| 1 | Expert Recommendation and References search |  | May 16, 2024 | 0 | 0 | 0 | 0 |
| 2 | PubMed | (((("aneurysm*"[Title/Abstract]) OR ("Intracranial aneurysm*"[Title/Abstract])) OR ("brain aneurysm*"[Title/Abstract])) OR ("cerebral aneurysm*"[Title/Abstract])) **AND** ("Woven Endobridge"[Title/Abstract]) OR ("WEB"[Title/Abstract]) **AND** (("retreatment"[Title/Abstract]) OR ("re-treatment"[Title/Abstract])) OR ("recurrence"[Title/Abstract]) | May 16, 2024 | 130 | 12 | 110 | 8 |
| 3 | Web of Science | ((((TS=("aneurysm*")) OR TS=("Intracranial aneurysm*")) OR TS=("brain aneurysm*")) OR TS=("cerebral aneurysm*") )**AND** ((TS=("retreatment")) OR TS=("re-treatment")) OR TS=("recurrence") **AND** (TS=(Woven Endobridge )) OR TS=("WEB") | May 16, 2024 | 136 | 18 | 109 | 9 |
| 4 | Scopus | ( TITLE-ABS-KEY ( "aneurysm*" ) OR TITLE-ABS-KEY ( "Intracranial aneurysm*" ) OR TITLE-ABS-KEY ( "brain aneurysm*" ) OR TITLE-ABS-KEY ( "cerebral aneurysm*" ) ) **AND** ( TITLE-ABS-KEY ( "Woven Endobridge" ) OR TITLE-ABS-KEY ( "WEB" ) ) **AND** ( TITLE-ABS-KEY ( "retreatment" ) OR TITLE-ABS-KEY ( "re-treatment" ) OR TITLE-ABS-KEY ( "recurrence" ) ) | May 16, 2024 | 185 | 13 | 162 | 10 |
| TOTAL |  |  |  | 451 | 43 | 384 | 24 |
